# Liver X Receptors Regulate the Transcriptional Activity of the Glucocorticoid Receptor: Implications for the Carbohydrate Metabolism

**DOI:** 10.1371/journal.pone.0026751

**Published:** 2012-03-22

**Authors:** Nancy Nader, Sinnie Sin Man Ng, Yonghong Wang, Brent S. Abel, George P. Chrousos, Tomoshige Kino

**Affiliations:** 1 Unit on Molecular Hormone Action, Program in Reproductive and Adult Endocrinology, Eunice Kennedy Shriver National Institute of Child Health and Human Development, National Institutes of Health, Bethesda, Maryland, United States of America; 2 School of Biomedical Science, Faculty of Medicine, The Chinese University of Hong Kong, Shatin, Hong Kong Special Administrative Region, People's Republic of China; 3 Microarray Facility, Advanced Technology Center, National Cancer Institute, National Institutes of Health, Gaithersburg, Maryland, United States of America; 4 First Department of Pediatrics, Athens University Medical School, Athens, Greece; Chinese University of Hong Kong, China

## Abstract

GLUCOCORTICOIDS are steroid hormones that strongly influence intermediary carbohydrate metabolism by increasing the transcription rate of glucose-6-phosphatase (G6Pase), a key enzyme of gluconeogenesis, and suppress the immune system through the glucocorticoid receptor (GR). The liver X receptors (LXRs), on the other hand, bind to cholesterol metabolites, heterodimerize with the retinoid X receptor (RXR), and regulate the cholesterol turnover, the hepatic glucose metabolism by decreasing the expression of G6Pase, and repress a set of inflammatory genes in immune cells. Since the actions of these receptors overlap with each other, we evaluated the crosstalk between the GR- and LXR-mediated signaling systems. Transient transfection-based reporter assays and gene silencing methods using siRNAs for LXRs showed that overexpression/ligand (GW3965) activation of LXRs/RXRs repressed GR-stimulated transactivation of certain glucocorticoid response element (GRE)-driven promoters in a gene-specific fashion. Activation of LXRs by GW3965 attenuated dexamethasone-stimulated elevation of circulating glucose in rats. It also suppressed dexamethasone-induced mRNA expression of hepatic glucose-6-phosphatase (G6Pase) in rats, mice and human hepatoma HepG2 cells, whereas endogenous, unliganded LXRs were required for dexamethasone-induced mRNA expression of phosphoenolpyruvate carboxylase. In microarray transcriptomic analysis of rat liver, GW3965 differentially regulated glucocorticoid-induced transcriptional activity of about 15% of endogenous glucocorticoid-responsive genes. To examine the mechanism through which activated LXRs attenuated GR transcriptional activity, we examined LXRα/RXRα binding to GREs. Endogenous LXRα/RXRα bound GREs and inhibited GR binding to these DNA sequences both in *in vitro* and *in vivo* chromatin immunoprecipitation assays, while their recombinant proteins did so on classic or G6Pase GREs in gel mobility shift assays. We propose that administration of LXR agonists may be beneficial in glucocorticoid treatment- or stress-associated dysmetabolic states by directly and gene-specifically attenuating the transcriptional activity of the GR on glucose and/or lipid metabolism.

## Introduction

GLUCOCORTICOIDS, steroid hormones produced by and secreted from the adrenal cortex, are essential for the maintenance of metabolic homeostasis both in the basal state and in response to stress [1], [2]. These hormones exert their actions in almost all tissues and organs, and strongly influence intermediary carbohydrate, lipid and protein metabolism [3]. For example, glucocorticoids induce gluconeogenesis by increasing the transcription rates of its key enzymes glucose-6-phosphatase (G6Pase), which mediates the final step of both gluconeogenesis and glycogenolysis [4] and phosphoenolpyruvate carboxykinase (PEPCK), which catalyzes the conversion of oxaloacetate to phosphoenolpyruvate [5], [6]. In addition to these metabolic effects, glucocorticoids also demonstrate strong suppressive effects on the immune system that makes them important therapeutic agents in the treatment of allergic, autoimmune, inflammatory and lymphoproliferative diseases [7]. However, chronic excess of glucocorticoid secretion, as occurs in endogenous Cushing syndrome and during chronic stress, or chronic administration of glucocorticoids for the treatment of responsive diseases, may lead to carbohydrate intolerance or frank diabetes, as well as to dyslipidemia (high VLDL- and LDL-cholesterol and low HDL-cholesterol), all detrimental conditions leading to atherosclerosis and cardiovascular diseases [8], [9], [10].

The biologic actions of circulating glucocorticoids are transmitted to the nucleus of cells by the ubiquitously expressed cytoplasmic/nuclear glucocorticoid receptor (GR), a member of the nuclear receptor superfamily that is comprised by over 130 proteins from nematodes to humans [3]. The human GR consists of 777 amino acids and has three major functional domains, the N-terminal (NTD) “immunogenic”, middle DNA-binding (DBD) and C-terminal ligand-binding (LBD) domains [11]. Upon hormone binding, the glucocorticoid-GR complex translocates from the cytoplasm into the nucleus and binds its specific DNA recognition sequences, the glucocorticoid response elements (GREs), located in the regulatory regions of glucocorticoid-responsive genes or interacts with other transcription factors to modulate the latter's transcriptional activities on their target genes by attracting numerous co-factor molecules and protein complexes to the respective transcription initiation complexes [11], [12].

The liver X receptors (LXRs), which also belong to the nuclear receptor superfamily, mediate the biologic actions of various lipids, such as the cholesterol metabolites oxysterols, and also prostanoids and some fatty acids, by directly binding to these molecules [13]. LXRs exist as two subtypes, LXRα and LXRβ, which display distinct patterns of tissue expression: LXRα is primarily expressed in the liver, intestine, adipose tissue, kidney and immune macrophages, whereas LXRβ is distributed ubiquitously [14]. Once LXRs bind their lipid ligands, they form a heterodimer with the retinoid X receptor (RXR), and stimulate the transcription of an array of genes involved in the absorption, efflux, transport, and excretion of cholesterol and other lipids [13], [14], [15]. LXRs also regulate glucose metabolism by decreasing the expression of its rate-limiting enzymes G6Pase and PEPCK [16], [17], [18], and have anti-inflammatory activity by repressing a set of inflammatory genes in macrophages and other immune cells [19]. These pieces of evidence indicate that the biologic actions of the GR and the LXRs may overlap with each other and that LXR activation may moderate the detrimental actions of chronically elevated glucocorticoids on carbohydrate and lipid metabolism. The purpose of this study is to evaluate the possible crosstalk between the GR- and LXR-mediated signaling systems by testing the effects of LXR activation on GR transcriptional activities and the ability of the former to moderate the effects of the latter.

## Results

### LXRs suppress dexamethasone-stimulated GR transcriptional activity on the MMTV promoter in HCT116 cells

**Figure 1:**
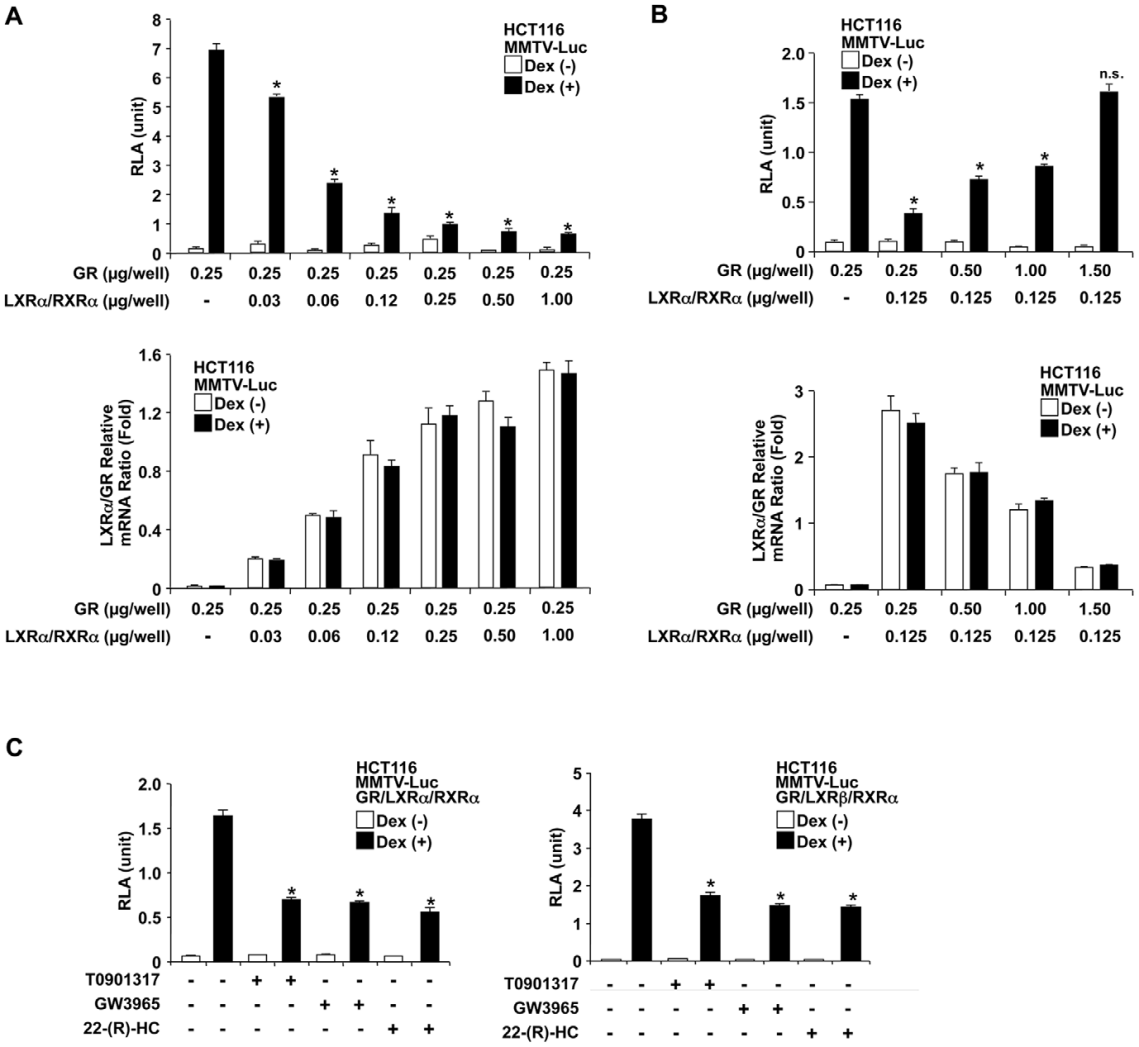
LXRs repress GR-induced transcriptional activity in HCT116 cells. A. Over-expression of LXRs strongly suppresses dexamethasone-stimulated transcriptional activity of the MMTV promoter in HCT116 cells. HCT116 cells were transfected with pRShGRα together with pMMTV-Luc and pGL4.73[*hRluc*/SV40], and increasing amounts of pCMX-hLXRα and pCMX-hRXRα (0.03–1 µg), and were incubated in the presence or absence of 10^−6^ M dexamethasone. *Upper panel:* Bars represent mean ± S.E. values of the firefly luciferase activity normalized for the renilla luciferase activity in the presence or absence of 10^−6^ M dexamethasone. *: p<0.01, n.s.: not significant, compared to the condition treated with dexamethasone in the absence of LXRα/RXRα. *Lower panel:* Total RNA from transfected HCT116 cells was harvested and relative mRNA expression of LXRα and GR were measured with the SYBR-Green real-time PCR. Bars represent mean ± S.E. values of the relative ratios of LXRα mRNA vs. GR mRNA using RPLP0 as an internal control. Dex: dexamethasone, RLA: relative luciferase activity. B. Over-expression of GR reverses the negative effect of LXRα/RXRα on dexamethasone-stimulated transcriptional activity of the MMTV promoter. HCT116 cells were transfected with increasing amounts of pRShGRα (0.25–1.5 µg) together with pMMTV-Luc, pGL4.73[*hRluc*/SV40], pCMX-hLXRα and pCMX-hRXRα, and were incubated in the presence or absence of 10^−6^ M dexamethasone. *Upper panel:* Bars represent mean ± S.E. values of the firefly luciferase activity normalized for the renilla luciferase activity in the presence or absence of 10^−6^ M dexamethasone. *: p<0.01, n.s.: not significant, compared to the condition treated with dexamethasone in the absence of LXRα/RXRα. *Lower panel:* Total RNA from transfected HCT116 cells was harvested and relative mRNA expression of LXRα and GR were measured with the SYBR-Green real-time PCR. Bars represent mean ± S.E. values of the relative ratios of LXRα vs. GR mRNA using RPLP0 as an internal control. Dex: dexamethasone, RLA: relative luciferase activity. C. Over-expression of LXRα (*left panel*) or LXRβ (*right panel*) in the presence of indicated LXR ligand suppresses dexamethasone-stimulated GR transcriptional activity on the MMTV promoter. HCT116 cells were transfected with pRShGRα, pMMTV-Luc and pGL4.73[*hRluc*/SV40] together with pCMX-hLXRα or -mLXRβ and pCMX-mRXRα, and were incubated in the presence or absence of 10^−6^ M dexamethasone and/or indicated LXR ligands. Bars represent mean ± S.E. values of the firefly luciferase activity normalized for the renilla luciferase activity. *: p<0.01 compared to the condition treated with dexamethasone in the absence of LXR ligand. Dex: dexamethasone, RLA: relative luciferase activity, 22-R-HC: 22-R-hydroxycholesterol.

To examine impact of LXRs on GR-mediated transcriptional activity, we first expressed human GR in HCT116 cells that do not express the endogenous molecule [20] together with the glucocorticoid-responsive mouse mammary tumor virus (MMTV) promoter-driven luciferase reporter gene (pMMTV-Luc), in the presence or absence of increasing amounts of the LXRα-expressing plasmid along with that of its obligate heterodimer partner RXRα. LXRα/RXRα over-expression strongly suppressed the transcriptional activity of the MMTV promoter in a dexamethasone-dependent fashion (Fig. 1A, *upper panel*). A significant decrease of 30% was observed in the presence of 0.03 µg of LXRα/RXRα-expressing plasmids, reaching up to 86% in the presence of 1 µg of plasmids expressing these heterodimer components. The observed negative effect was totally reversed by increasing amounts of the GR-expressing plasmid, suggesting that there may be competition between LXRα/RXRα and GR (Fig. 1B, *upper panel*). In these experiments, we measured the relative mRNA expression of LXRα and GR using the acidic ribosomal phosphoprotein P0 (RPLP0) as an internal control, calculated relative ratios of LXRα mRNA vs. GR mRNA, and confirmed that the transcriptional changes observed were due to alteration in the expressions of these NRs (Fig. 1A and 1B, *lower panels*). The suppressive effect of LXRα/RXRα on dexamethasone-stimulated transcriptional activity of the MMTV promoter was observed in the absence of LXR-specific ligands (Fig. 1 A and B, *upper panels*), consistent with a previous report that LXRs are activated just by heterodimerization in the absence of ligand, via a mechanism termed ‘dimerization-induced activation’ [21]. This is a unique activation mechanism for LXRs, which has not been described for the other nuclear receptors [21]. We further tested the effect of LXR ligands on GR-induced transcriptional activity, by over-expressing LXRα or LXRβ along with RXRs in HCT116 cells and by treating these cells with or without different LXR ligands, such as the natural ligand 22-R hydroxycholesterol (22-R-HC) and the two synthetic ligands T0901317 and GW3965. T0901317 activates LXRs but also other nuclear receptors, such as the pregnane X receptor (PXR), whereas GW3965 is a specific ligand for LXRs [22], [23]. Activation of over-expressed LXRα (Fig. 1C, *left panel*) or β (Fig. 1C, *right panel*) by these ligands enhanced LXR/RXR-mediated repression of GR-induced transcriptional activity on the MMTV promoter by more than 50% (Fig. 1C).

### LXRα regulates GR-mediated transcriptional activity in a gene promoter-specific fashion

**Figure 2:**
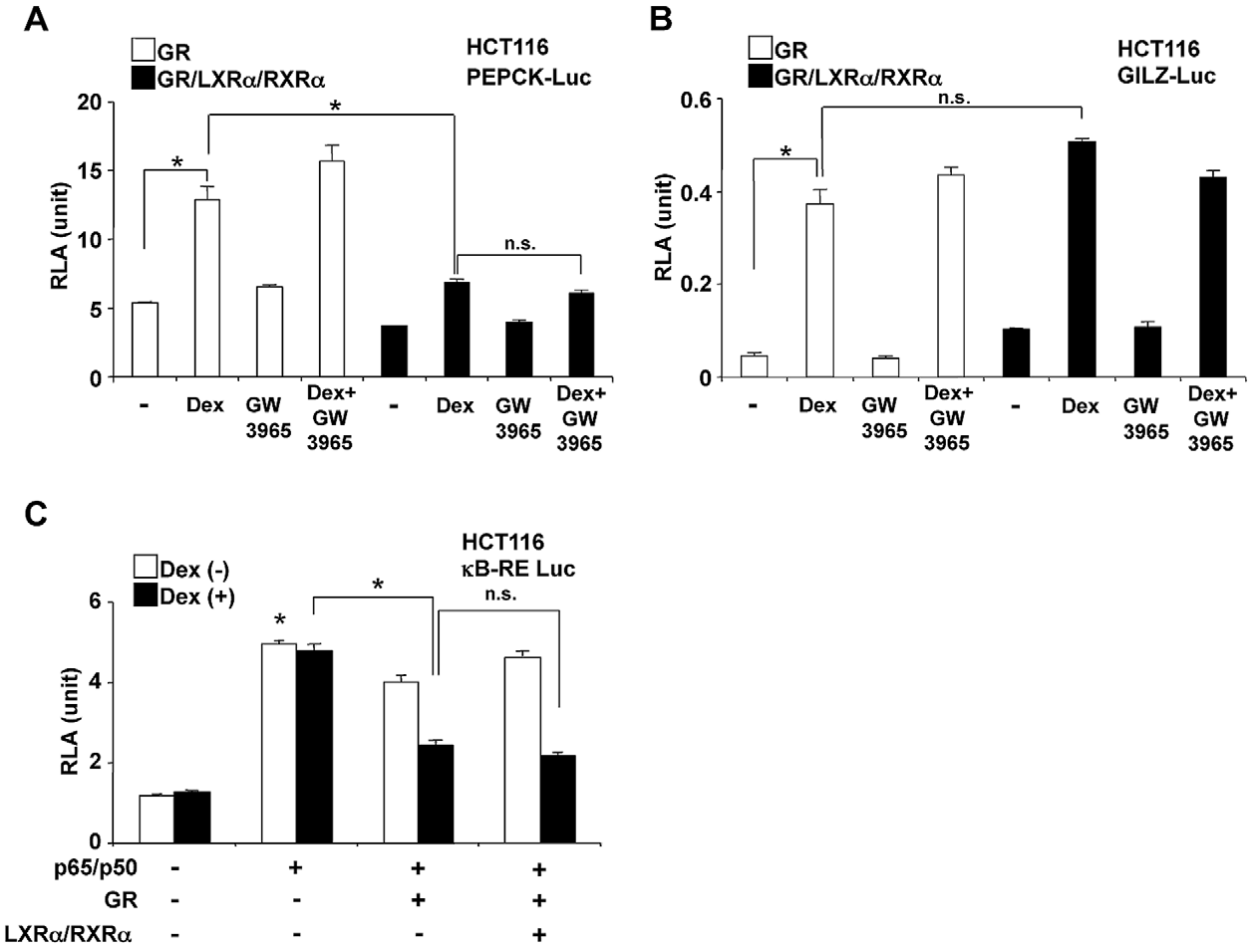
Over-expression of LXRα regulates dexamethasone-stimulated GR transcriptional activity in a gene promoter-specific way. A and B. Over-expression of LXRα/RXRα suppresses dexamethasone-stimulated transcriptional activity of the PEPCK (A) but not the GILZ (B) promoter. HCT116 cells were transfected with pPEPCK-luc (A) or pGILZ-Luc (B), together with pGL4.73[*hRluc*/SV40] and pRShGRα in the presence or absence of pCMX-hLXRα and pCMX-hRXRα, and were incubated with or without 10^−6^ M dexamethasone and/or 10^−6^ M GW3965. Bars represent mean ± S.E. values of the firefly luciferase activity normalized for the renilla luciferase activity. *: p<0.01, n.s.: not significant between the two conditions indicated. Dex: dexamethasone, RLA: relative luciferase activity. C. Over-expression of LXRα/RXRα does not affect dexamethasone-induced repression of the transcriptional activity of NF-κB. HCT116 cells were transfected with (κB)_3_-Luc and pGL4.73[*hRluc*/SV40] in the presence or absence of pRSV-RelA (p65)/pRSV-NF-κBI (p50), pRShGRα, and pCMX-hLXRα and pCMX-hRXRα, and were incubated with or without 10^−6^ M dexamethasone. Bars represent mean ± S.E. values of the firefly luciferase activity normalized for the renilla luciferase activity. *: p<0.01, n.s.: not significant, compared to the condition obtained in the absence of p65/p50 and dexamethasone treatment, or between the two conditions indicated. Dex: dexamethasone, RLA: relative luciferase activity.

We next investigated the effect of LXRα on the transactivational activity of GR on two glucocorticoid-responsive genes, the phosphoenolpyruvate carboxykinase (*PEPCK*) and the glucocorticoid-inducible leucine zipper protein (*GILZ*), both of which contain functional GREs within their promoter regions [6], [24]. We transfected HCT116 cells with GR-expressing plasmid along with the PEPCK or GILZ promoter-driven luciferase reporter, pPEPCK-Luc (Fig. 2A) or pGILZ-Luc (Fig. 2B), in the presence or absence of LXRα/RXRα over-expression. GR stimulated the transcriptional activity of these promoter constructs in a dexamethasone-dependent fashion. Overexpression of LXRα/RXRα repressed dexamethasone-stimulated transcriptional activity of the PEPCK promoter by approximately 50%, with no significant change after addition of GW3965 (Fig. 2A), while no significant suppressive effect was observed on dexamethasone-stimulated transcriptional activity of the GILZ promoter (Fig. 2B). We further examined the effect of LXRα/RXRα on the transrepressive effect of the GR (Fig. 2C) on a nuclear factor κB (NF-κB)-responsive reporter gene in HCT116 cells by over-expressing the NF-κB components p65 and p50 in the presence of the κB-response element (RE)-driven reporter plasmid. Overexpression of p65/p50 stimulated the activity of this promoter, while dexamethasone suppressed it in a GR expression-dependent fashion. Overexpression of LXRα/RXRα did not influence GR-mediated repression of p65/p50-induced transcriptional activity (Fig. 2C), suggesting that LXRα/RXRα does not affect the transrepressive effect of GR caused via physical interaction with other transcription factors. Taken together, these findings indicate that LXRα/RXRα affect the transactivating, but not the transrepressive activity of the GR in a gene-specific fashion in HCT116 cells.

### LXRs differentially regulate dexamethasone-induced mRNA expression of endogenous glucocorticoid-responsive genes in HepG2 cells

**Figure 3:**
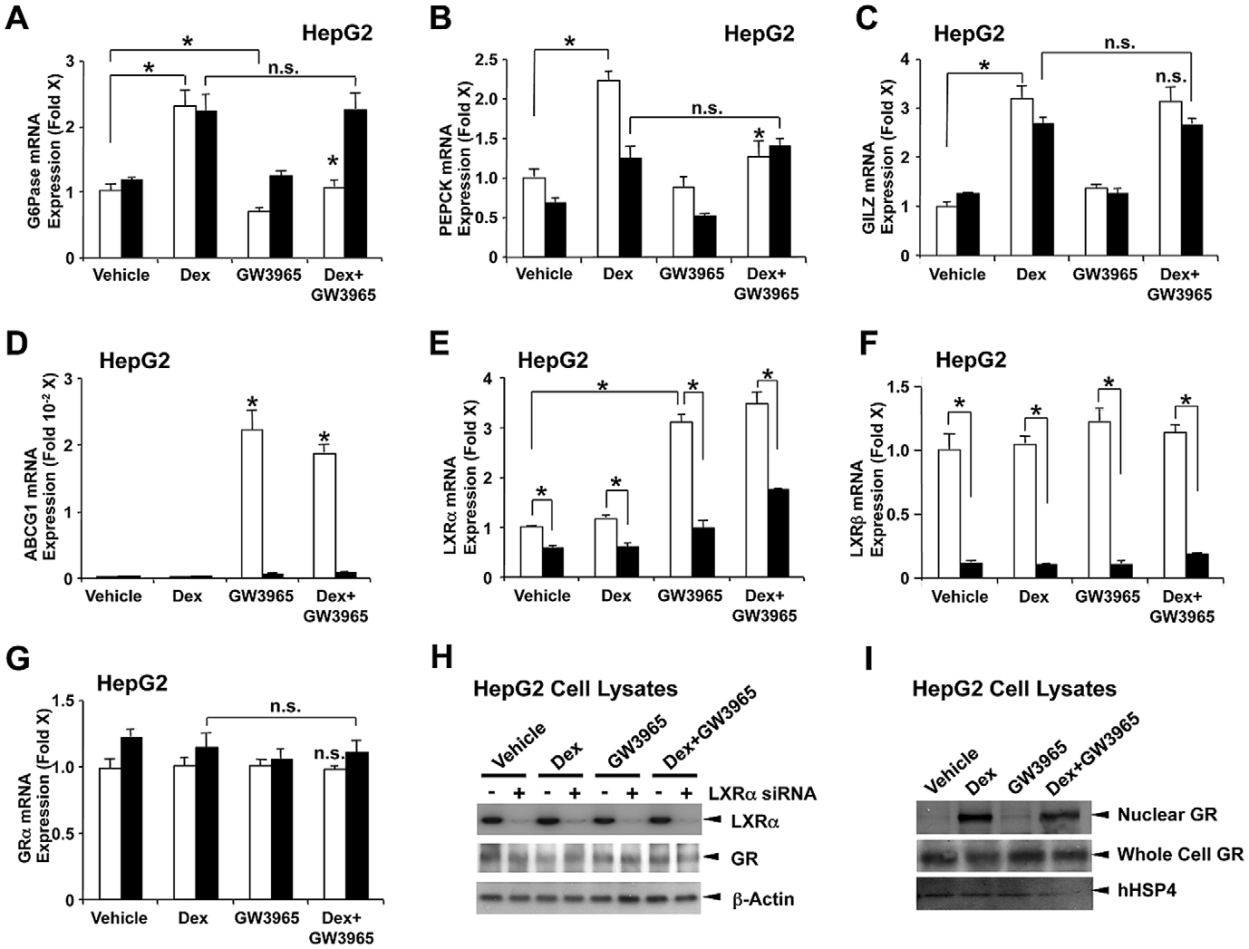
Ligand activation of endogenous LXRs differentially regulates dexamethasone-induced mRNA expression of glucocorticoid-responsive genes in a gene-specific fashion. A, B, C, D, E, F and G. HepG2 cells were transfected with negative control siRNA for luciferase GL2 (white bars) or siRNAs for LXRα/β (black bars), and were treated with or without 10^−6^ M dexamethasone and/or 10^−6^ M GW3965. Total RNA was harvested and mRNA levels of G6Pase (A), PEPCK (B), GILZ (C), ABCG1 (D), LXRα (E), LXRβ (F) and GR (G) were measured with the SYBR-Green real-time PCR. Bars represent mean ± S.E. values of the fold induction of mRNA expression. *: p<0.01, n.s.: not significant compared to the condition transfected with the control siRNA in the presence of dexamethasone or between the two conditions indicated. Dex: dexamethasone. H. siRNAs for LXRα suppress expression of LXRα protein in HepG2 lysates. HepG2 cells were transfected with negative control siRNA for luciferase GL2 or siRNAs for LXRα, and were treated with or without 10^−6^ M dexamethasone and/or 10^−6^ M GW3965 for 24 hours, and cells lysates were prepared. hLXRα (*upper panel*), hGR (*middle panel*) and hβ-actin (*lower panel*) were visualized with their specific antibodies in Western lots. I. Ligand activation of endogenous LXRs does not influence dexamethasone-induced translocation of GR from the cytoplasm into the nucleus. HepG2 were treated for 2 hours with or without 10^−6^ M dexamethasone and/or 10^−6^ M GW3965. Whole cell lystates and nuclear extracts were run on 4–20% SDS-PAGE gels, blotted to nitrocellulose membranes and hGR (*upper panel*: nuclear GR, *middle panel*: whole cell GR) and hHSP4 (*lower panel*) were visualized with their specific antibodies in Western blots.

We next examined the effect of specific LXR ligand GW3965 on dexamethasone-stimulated mRNA expression of endogenous glucocorticoid-responsive genes in HepG2 cells. GW3965 completely attenuated dexamethasone-induced mRNA expression of the *G6Pase* (Fig. 3A, white bars) and *PEPCK* (Fig. 3B, white bars) genes, while no effect was observed on dexamethasone-induced mRNA expression of the *GILZ* gene (Fig. 3C, white bars). When GW3965 was added, we observed a slight but significant decrease of *G6Pase* mRNA expression (Fig. 3A, white bars), as well as a significant increase of *LXRα* mRNA expression (Fig. 3E, white bars) consistent with previous reports [17], [25]. GW3965 strongly increased mRNA expression of the LXR-responsive ATP-binding cassette sub-family G member 1 (*ABCG1*) gene [26], indicating that this compound efficiently activated LXRs (Fig. 3D, white bars). To demonstrate the specificity of the observed GW3965 effect, we knocked down the expression of both *LXRα* and *LXRβ* with their respective siRNAs (Fig. 3, black bars). Transfection of HepG2 cells with these siRNAs efficiently suppressed mRNA and protein expression of *LXRα* and *β* (Fig. 3E and 3F, black bars, and 3H), and completely abolished GW3965-induced suppression of dexamethasone-mediated mRNA expression of the *G6Pase* and *PEPCK* genes (Fig. 3A and 3B, black bars), indicating that activation of LXRs by GW3965 directly mediates the negative effect of this compound on GR-induced transcriptional activity on the endogenous *G6Pase* and PEPCK genes. In contrast, LXR*α*/*β* knockdown in itself attenuated dexamethasone-stimulated mRNA expression of the *PEPCK* gene (Fig. 3B, black bars), suggesting that endogenous LXRs are required for the optimum action of dexamethasone on this gene in the absence of GW3965. GW3965 did not alter mRNA expression of *GR* throughout the experiment (Fig. 3G), and Western blots for LXRα and GR using lysates obtained from HepG2 cells treated with vehicle, Dex and/or GW3965 showed that transfection of HepG2 cells with LXRα siRNA efficiently suppressed LXRα protein levels but not those of the GR (Fig. 3H). In addition, Western blots for GR using nuclear extracts of HepG2 cells treated with GW3965 and/or dexamethasone demonstrated that GW3965 did not influence dexamethasone-induced accumulation of GR in the nucleus (Fig. 3I). These results further indicate that agonist-activated endogenous LXRs suppress GR-induced transcriptional activity on natural glucocorticoid-responsive genes in a gene-specific fashion in addition to the artificial reporter assay system in which LXRs, GR and glucocorticoid-responsive promoters were exogenously introduced. On the other hand, unliganded LXRs are required for GR to stimulate mRNA expression of some glucocorticoid-responsive genes. Our results also suggest that LXRs influence the transcriptional activity of GR inside the nucleus via a molecular mechanism that does not include modification of GR expression and nuclear translocation.

### GW3965 attenuates dexamethasone-induced elevation of circulating glucose levels in rats and suppresses G6Pase mRNA expression by this steroid in rat and mouse livers

**Figure 4:**
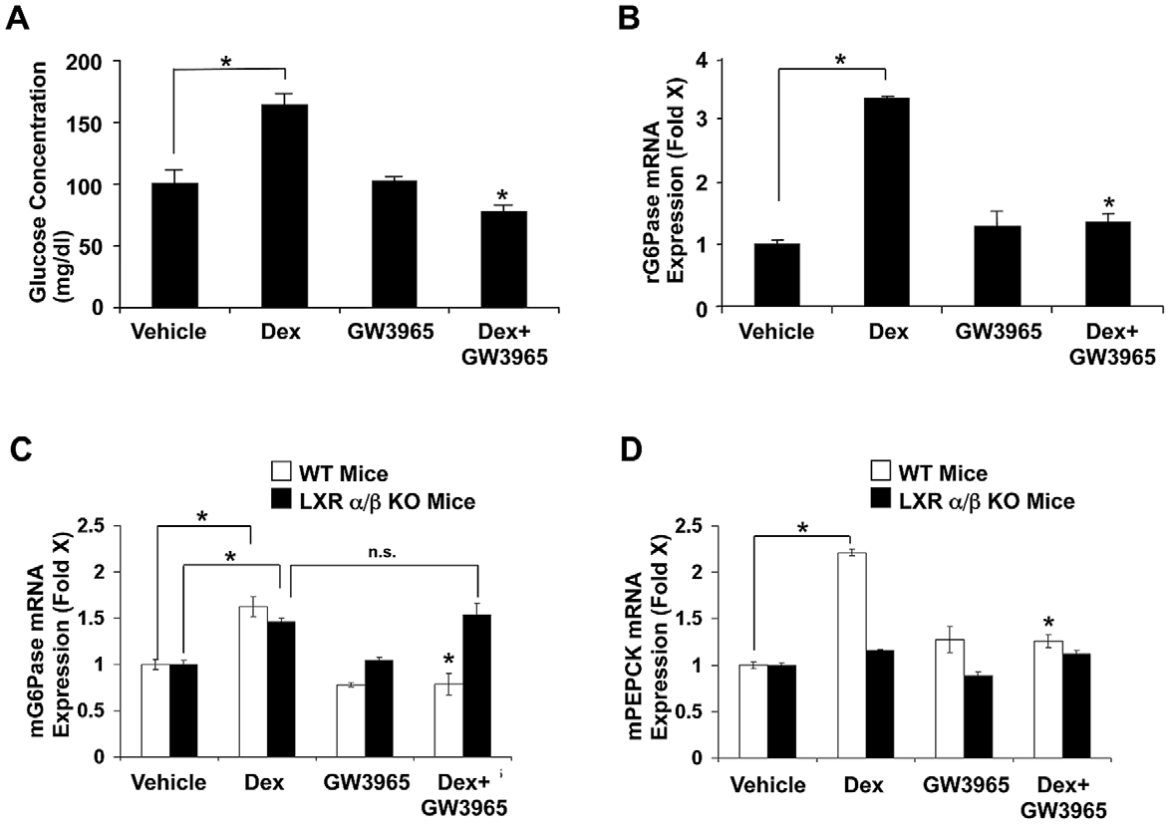
GW3965 attenuates dexamethasone-induced hyperglycemia in rats and represses dexamethasone-induced G6Pase mRNA expression in mouse and rat livers and PEPCK mRNA expression in mouse livers. A. GW3965 attenuates dexamethasone-induced increase of blood glucose levels in rats. Blood glucose levels were measured in rats treated with the compounds indicated. Bars represent mean ± S.E. values of the blood glucose concentration. *: p<0.01, compared to the animals treated with dexamethasone or between the two conditions indicated. Dex: dexamethasone. B, C and D. GW3965 suppresses dexamethasone-induced mRNA expression of glucocorticoid-responsive *G6Pase* in the livers of rats (B), *G6Pase* and *PEPCK* in wild type, but not LXRα/β knockout mice (C and D). Rats, wild type (WT) and LXRα/β knockout mice were orally gavaged for three days with GW3965, and were injected with dexamethasone or physiologic saline intramuscularly. Twenty-four hours after the injection, Total RNA was harvested from their livers and mRNA levels of rat *G6Pase* (B), mouse *G6Pase* (C) and mouse *PEPCK* (D) were measured with the SYBR-Green real-time PCR. Bars represent mean ± S.E. values of the fold induction of mRNA expression against vehicle. *: p<0.01, n.s.: not significant, compared to the animals treated with dexamethasone or between the two conditions indicated. Dex: dexamethasone, KO: knockout.

Since LXRs strongly suppressed GR-induced mRNA expression of the *G6Pase* and *PEPCK* genes in HepG2 cells, and they play a key role in glucose metabolism, we examined the effect of GW3965 on dexamethasone-induced hyperglycemia in rats, as well as *G6Pase* mRNA expression in rat and mouse livers. Dexamethasone increased blood glucose levels by ∼40% in rats after 24 hours of the injection, while GW3965 strongly prevented the elevation caused by this steroid (Fig. 4A). GW3965 completely attenuated dexamethasone-induced mRNA expression of G6Pase in the rat livers (Fig. 4B). Importantly, LXRα/β knockout mice lost GW3965-induced suppression of G6Pase mRNA induction by dexamethasone in the liver, while wild type mice demonstrated a GW3965-mediated suppression similar to that of rats (Fig. 4B and 4C). GW3965 completely attenuated dexamethasone-induced mRNA expression of the *PEPCK* gene in the livers of wild type mice, while LXRα/β knockout mice lost dexamethasone-stimulated mRNA expression of this gene in the absence of GW3965 (Fig. 4D). These results are consistent with our findings obtained in HepG2 cells transfected with LXRα/β siRNAs, further confirming that endogenous LXRs have dual effects on GR-stimulated mRNA expression of the *PEPCK* gene. Taken together, these results indicate that LXRs repress GR-induced transcriptional activity at the animal level, attenuating elevations of circulating glucose levels and suppressing expression of liver *G6Pase* and *PEPCK* mRNA stimulated by glucocorticoids.

### LXRs differentially regulate GR-induced transcriptional activity in rat liver in microarray analysis

**Figure 5:**
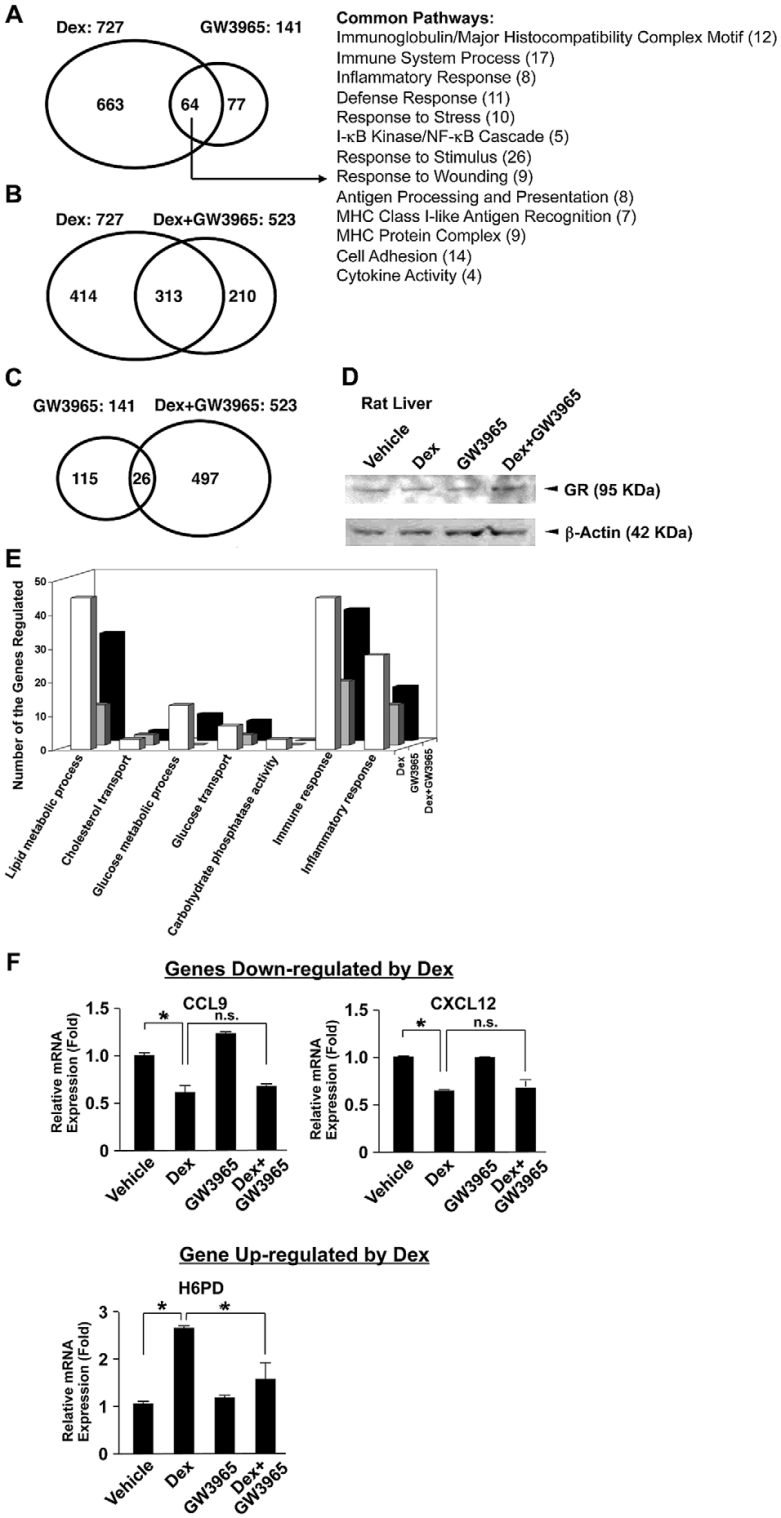
GW3965 alters dexamethasone-induced mRNA expression of its responsive genes in rat liver in microarray analysis. A, B and C: GW3965 modulates the transcriptional activity of a fraction of dexamethasone-responsive genes. Venn diagrams demonstrating the number of genes regulated by dexamethasone (Dex) and/or GW3965 (A), Dex and/or Dex+GW3965 (B) and GW3965 and/or Dex+GW3965 (C). The common biologic pathways regulated independently by Dex or GW3965 are also demonstrated in the right side of panel A. D. The 72-hour GW3965 treatment does not alter GR protein levels in the rat liver. Tissue lysates were prepared from rat livers treated with indicated compounds. GR (*upper panel*) and β-actin (*lower panel*) were visualized with their specific antibodies in Western blots. E. The number of genes regulated by Dex and GW3965 in respective biologic pathways is shown. Nomenclatures of the demonstrated biologic activities are based on the ontology of the Gene Ontology: http://www.geneontology.org. F. GW3965 does not influence mRNA expression of dexamethasone-suppressive genes in rat livers. Total RNA was harvested from rat livers and mRNA levels of *Ccl9* (*left upper panel*), *Cxcl12* (*right upper panel*) and *H6pd* (*lower panel*) were measured with the SYBR-Green real-time PCR. Bars represent mean ± S.E. values of the fold induction of mRNA expression. *: p<0.01, n.s.: not significant between the two conditions indicated. Dex: dexamethasone.

To further address functional interaction between LXRs and GR at a transcriptome level, we performed microarray analysis using the Rat Genome 230 2.0 Tiled Array (Affymetrix Inc.) in samples obtained from livers of rats treated with dexamethasone and/or GW3965 (Fig. 5). Among ∼15,000 genes successfully analyzed, dexamethasone changed mRNA expression of 727 (∼4.8%) genes, and GW3965 altered the expression of 141 genes (∼1%) (Fig. 5A), among which 77 genes were dexamethasone unresponsive (Table S1). Dexamethasone and GW3965 shared 64 genes that are mostly implicated in immune and inflammatory response pathways (Fig. 5A). Simultaneous treatment with dexamethasone and GW3965 (Dex+GW3965) changed mRNA expression of 523 genes implicated in different biological pathways, among which 313 genes were also regulated by dexamethasone alone (Fig. 5B). GW3965 treatment did not alter mRNA expression of 202 genes out of these 313 common genes, whereas the remaining 111 genes (∼15% of the total 727 dexamethasone-regulated genes) showed an expression that was significantly different from the dexamethasone treatment alone, indicating that GW3965 influenced dexamethasone-induced changes in these 111 genes. Of these 111 genes, GW3965 treatment altered mRNA expression of 45 (∼6%) genes down-regulated by dexamethasone, and 66 (∼9%) genes up-regulated by this steroid, indicating that GW3965 influences GR-induced transactivation more frequently than transrepression. Furthermore, GW3965 treatment attenuated dexamethasone-induced suppressive and enhancing effect on the mRNA expression of the majority of the 111 genes (Tables S2 and S3). Numbers of the genes in some specific biological pathways regulated by dexamethasone and/or GW3965 are also shown in Fig. 5E.

In contrast to the effect of GW3965 on GR-induced transcriptional activity, only 26 genes out of 141 GW3965-responsive genes were influenced by the addition of dexamethasone (Fig. 5C), suggesting that in the rat liver the GR influence on LXR actions is not as strong as that of LXRs on GR actions. To examine whether the 72-hour GW3965 treatment alters GR protein levels in the rat liver, we performed Western blots for GR in the liver of rats treated for 72 hours, and found that GW3965 did not alter GR protein levels (Fig. 5D). . These results rule out the possibility of GW3965 altering dexamethasone-induced gene expression by affecting GR expression levels

In Table 1, we summarized the gene-selective effects of LXRs on GR-induced transcriptional activity. We cited some of the genes known to be associated with the transrepressive activity of the GR, such as the stem cell factor (*Scf*), chemokine (C-C motif) ligand 9 (*Ccl9*), interleukin 33 (*Il33*) and chemokine (C-X-C motif) ligand 12 (*Cxcl12*) genes, which encode proteins that belong to the family of cytokines involved in immunoregulatory and inflammatory processes, and are often induced by proinflammatory stimuli [27], the cyclin D1 gene (*Ccnd1*), a key regulator of cell proliferation whose overexpression plays a role in tumorigenesis [28], and the cytokine-inducible SH2-containing protein (*Cish*), which belongs to the cytokine-induced STAT inhibitor (CIS) family, whose members are induced by cytokines and act as negative regulators of cytokine signaling [29]. Dexamethasone down-regulated mRNA expression of all these genes, while GW3965 did not significantly influence the suppressive effect of dexamethasone on the mRNA expression of the *Scf*, *Ccl9*, *Il33*, or *Cxcl12* genes; In contrast, GW3965 reversed the negative effect of dexamethasone on the mRNAs of the *Ccnd1* and *Cish* genes. Dexamethasone up-regulated mRNA expression of the 6-phosphofructo-2-kinase/fructose-2,6-biphosphatase 1 (*Pfkfb1*) gene, which encodes a protein functioning as an activator of glycolysis and an inhibitor of gluconeogenesis, the *Slc2a5* gene encoding the glucose/fructose transporter protein member 5 (GLUT5) that regulates glucose and fructose transport from the intestinal lumen into the enterocytes [30], [31], the *G6Pase* gene, the hexose-6-phosphate-dehydrogenase (*H6pdh*) gene, encoding an enzyme generating reduced nicotinamide adenine dinucleotide phosphate (NADPH) that is required by 11β-hydroxysteroid dehydrogenase type 1 (11β-HSD1) for the activation of glucocorticoids [32]. Activated GR also increased mRNA expression of the lipin 1 (*Lpin1*) and serine dehydratase (*Sds*) genes, as previously reported [33]: The former encodes a member of the lipin enzyme family that plays a role in human triglyceride metabolism and influences glucose homeostasis in diverse organs and tissues, including the liver [34], while the latter gene product converts serine to pyruvate and is involved in gluconeogenesis [35]. GW3965 completely suppressed dexamethasone-induced mRNA expression of *G6Pase* and *H6pd*, and significantly repressed that of *Lpin1* and *Sds*, while it did not affect dexamethasone-mediated induction of *Pfkfb1* and *Slc2a5* mRNA. In contrast, GW3965 significantly enhanced dexamethasone-induced mRNA expression of the interferon-induced protein 44 (Ifi44) and the 2′-5′ oligoadenylate synthetase 1A (Oas1a), both of which play key roles in innate immune responses against viral infections [36], [37]. We confirmed our microarray results by examining in the reconstituted real-time PCR assays mRNA expression of the *Ccl9* and *Cxcl12* genes, as representatives of the genes down-regulated by dexamethasone (Fig. 5F, *upper panel*), and the *H6pd* gene, as a representative of genes up-regulated by this steroid (Fig. 5F, *lower panel*). Taken together, these results are consistent with our findings obtained in transient transfection-based reporter assays that LXRs regulate GR-transcriptional activity in a gene-specific fashion.

**Table 1:** mRNA Expression of Selective Glucocorticoid-responsive Genes in the Liver of Rats Treated with Dexamethasone in the Absence or Presence of GW3965.

Protein	Gene symbol	Log ratio Dex*	Log ratio Dex+GW3965*
Stem cell factor	*Scf*	−2.350	−2.639
Chemokine (C-C motif) ligand 9	*Ccl9*	−1.141	−1.172
Interleukin 33	*Il33*	−2.402	−2.282
Chemokine (C-X-C motif) ligand 12	*Cxcl12*	−2.205	−1.599
Cyclin D1	*Ccnd1*	−2.435	−1.131
Cytokine inducible SH2-containing protein	*Cish*	−2.008	Not significant
6-Phosphofructo-2-kinase/fructose-2,6-biphosphatase 1	*Pfkfb1*	2.244	1.732551669
Solute carrier family 2 (facilitated glucose/fructose transporter), member 5	*Slc2a5*	1.155	1.336
Glucose-6-phosphatase	*G6pc*	1.473	Not significant
Hexose-6-phosphate dehydrogenase (glucose-1-dehydrogenase)	*H6pd*	1.206	Not significant
Serine dehydratase	*Sds*	3.521152	1.778876
Lipin 1	*Lpin1*	3.052642	1.49767
Interferon-induced protein 44	*Ifi44*	1.945108	2.775536
2′-5′ Oligoadenylate synthetase 1A	*Oas1a*	3.992291	4.477496

Dex: dexamethasone.

* : Results demonstrated were sorted from those of the microarray analyses.

### LXRα/RXRα heterodimer competes with GR for binding to GREs

**Figure 6:**
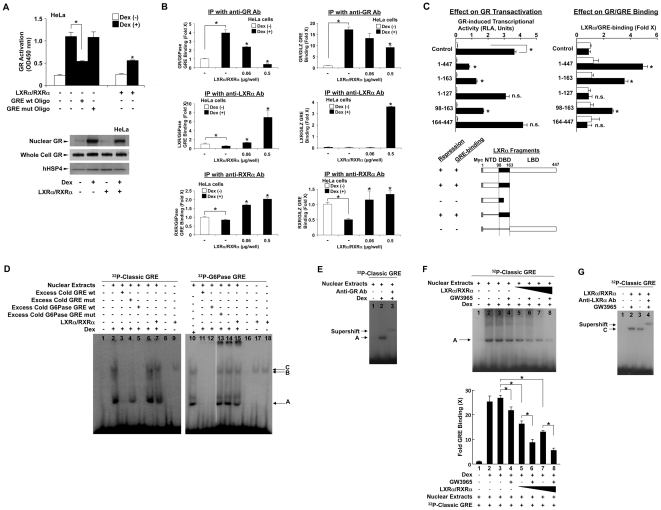
LXRα/RXRα attenuates the association of GR to GREs *in vitro* and *in vivo*. A. *Upper panel*: LXRα/RXRα suppresses association of the GR to its classic consensus GRE *in vitro*. HeLa cells were transfected with pCMX-hLXRα and pCMX-hRXRα, or their carrying plasmid pCMX, and were treated with or without 10^−6^ M dexamethasone for 2 hours. Nuclear extracts were harvested from the cells and the binding activity of GR to GREs was evaluated in the presence or absence of wild type (wt) or mutant (mut) GRE oligonucleotides (Oligos) using the TRansAM™ GR kit (Active Motif). Bars represent mean ± S.E. values of the absorbance at 450 nm in the presence or absence of 10^−6^ M of dexamethasone. *: p<0.01, compared to the condition transfected with the control plasmid in the presence of dexamethasone and in the absence of GRE Oligos or between the two conditions indicated. Dex: dexamethasone. *Lower panel*: Overexpression of LXRα/RXRα does not influence dexamethasone-induced translocation of GR from the cytoplasm into the nucleus. Nuclear extracts obtained from HeLa cells were run on 4–20% SDS-PAGE gels, blotted to nitrocellulose membranes and GR (*upper panel*: nuclear GR, *middle panel*: whole cell GR) and hHSP4 (*lower panel*) were visualized with their specific antibodies in Western blots. B. LXRα/RXRα overexpression differentially affects the association of GR to its GREs in ChIP assays. HeLa cells were transfected with the empty control pCMX vector, or pCMX-hLXRα and -hRXRα, treated with or without 10^−6^ M dexamethasone, and ChIP assays were performed using anti-hGRα, anti-hLXRα and anti-hRXRα antibodies. Bars represent mean ± S.E. values of the fold precipitation of G6Pase GREs (*left panel*) or GILZ GREs (*right panel*) determined in the SYBR Green-based real-time PCR. *: p<0.01, n.s.: not significant, compared to the condition transfected with the control plasmid in the presence of dexamethasone or between the two conditions indicated. Dex: dexamethasone, IP: immunoprecipitation. C. DBD of LXRα confers LXRα-mediated repression of GR transcriptional activity and binding to G6Pase GREs. HCT116 cells were transfected with pRShGRα together with pMMTV-Luc and pGL4.73[*hRluc*/SV40], pCMX-hRXRα and the pCDNA3-6myc plasmid expressing wild type LXRα (1–447) or indicated LXRα mutants. Cells were incubated in the presence (black columns) or absence (white columns) of 10^−6^ M dexamethasone. Using aliquots of cell lysates, luciferase assays were performed. ChIP assays were also performed by treating cells with 4% formaldehyde, and by precipitating LXRα/G6Pase GREs with anti-Myc-antibody. Bars represent mean ± S.E. values of the firefly luciferase activity normalized for the renilla luciferase activity (*left panel*) or of the fold precipitation of G6Pase GREs (*right panel*) determined in the SYBR Green-based real-time PCR in the presence or absence of 10^−6^ M dexamethasone. *: p<0.01, n.s.: not significant, compared to the condition treated with dexamethasone in the absence of LXRα/RXRα or between the two conditions indicated. NTD: N-terminal domain, DBD: DNA-binding domain, LBD: ligand-binding domain. D, E, F, G. LXRα/RXRα heterodimer binds to classic consensus and G6Pase GREs in gel mobility shift assays and reduces GR binding to its GREs. D. Nuclear extracts from HeLa cells or LXRα/RXRα recombinant proteins were incubated with ^32^P-labeled classic consensus GRE (*left panel*) or G6Pase GRE (*right panel*) with dexamethasone (Dex) in the presence or absence of an excess amount (50-fold molar excess) of unlabeled classic consensus GRE wild type (wt), GRE mutated (mut), G6Pase GRE wt, or G6Pase GRE mut. E. Nuclear extracts from HeLa cells were incubated with ^32^P-labeled classic consensus GRE in the presence or absence of dexamethasone (Dex) and in the presence or absence of anti-GR antibody (Anti-GR Ab). F. Nuclear extracts from HeLa cells were incubated with ^32^P-labeled classic consensus GRE in the presence or absence of dexamethasone (Dex) and/or GW3965 under the presence of increased amounts of LXRα/RXRα recombinant proteins (*upper panel*). The intensities of band A from 3 independent experiments including that shown in the upper panel were measured with the image J software and fold GRE binding was calculated by comparing to control (lane 1) (*lower panel*). Bars represent mean ± S.E. values of fold GRE binding from 3 independent experiments. *: p<0.01, compared to the conditions indicated. G. LXRα/RXRα recombinant proteins were incubated with ^32^P-labeled classic consensus GRE in the presence or absence of GW3965 and anti-LXRα antibody (Anti-LXRα Ab).

To examine the mechanism(s) underlying LXR-induced repression of GR transcriptional activity, we examined the effect of LXRα/RXRα overexpression on the binding of GR to its DNA recognition sequences GREs *in vitro* and *in vivo* (Fig. 6), granted that we noticed in the transfection experiment that LXRα/RXRα and GR competed with each other for dexamethasone-mediated activation of the MMTV promoter (Fig. 1). In our *in vitro* GR/GRE binding assay, nuclear GR extracted from HeLa cells bound to the classic consensus immobilized GRE oligonucleotide in a dexamethasone-dependent fashion, while free oligonucleotides encoding wild type, but not mutated, GREs inhibited this binding (Fig. 6A). Over-expression of LXRα/RXRα in these cells attenuated dexamethasone-mediated association of GR to immobilized GREs (Fig. 6A, *upper panel*). Overexpression of LXRα/RXRα did not influence dexamethasone-induced accumulation of GR in the nucleus in the samples used in our *in vitro* GR/GRE-binding assay (Fig. 6A, *lower panel*). We next examined the effect of over-expressed LXRα/RXRα on the association of GR to endogenous G6Pase and GILZ GREs in chromatin immunoprecipitation (ChIP) assays (Fig. 6B). In HeLa cells, GR bound to G6Pase and GILZ GREs in a dexamethasone-dependent fashion. Over-expression of LXRα along with RXRα strongly inhibited this dexamethasone-mediated association of GR to G6Pase GREs in a dose-dependent fashion (Fig. 6B, left *upper panel*), while it demonstrated a weaker suppressive effect on the association of GR to GILZ GREs, also in a dose-dependent fashion (Fig. 6B right *upper panel*). When we used anti-LXRα antibody for pull-down of protein/DNA complexes, some background precipitation of LXRα to G6Pase, but not GILZ, GREs was observed in the absence of dexamethasone (Fig. 6B, left and right *middle panels*, white bars). RXRα was more obviously co-precipitated both with G6Pase and GILZ GREs by anti-RXRα antibody in the absence of dexamethasone (Fig. 6B, left and right *lower panels*, white bars). Dexamethasone weakly attenuated co-immunoprecipitation of LXRα or RXRα with G6Pase GREs, while it strongly suppressed RXRα co-immunoprecipitation with GILZ GREs. Over-expression of LXRα/RXRα competed with dexamethasone-induced inhibition of their binding with G6Pase and GILZ GREs (Fig. 6B, left and right *middle* and *lower panels*). Pull-down with negative control IgG did not show any difference in the precipitation of G6Pase or GILZ-GREs throughout the experiment (data not shown). We also found association of LXRα and RXRα on G6Pase, but not GILZ, GREs in HepG2 cells (data not shown), consistent with our results demonstrating the suppressive effect of LXRα/RXRα on GR transcriptional activity in these cells (Fig. 3).

Our data suggesting that LXRα might impair GR activity by competing with GR for GRE binding highlights a probable role of the LXRα DBD. Thus, we examined the effect of a series of LXRα mutants on GR-induced transcriptional activity in HCT116 cells together with their binding to G6Pase GREs in ChIP assays. Expression of such LXR mutants from the plasmids employed was previously reported [38]. We found that the repressive effect of LXRα on GR-induced transcriptional activity was associated with the presence of its DBD, while this domain of LXRα was critical for binding to the GREs (Fig. 6C), These results indicate that LXRα competes with GR for binding to GREs and subsequent activation of transcription via its DBD.

To further investigate the mechanism by which LXRα/RXRα reduce the binding of GR to GREs, we performed gel mobility shift assays using nuclear extracts from HeLa cells and ^32^P-radiolabeled classic consensus GRE wild type (wt) or G6Pase GRE wt (Fig. 6D, *left and right panels*, respectively). Under the dexamethasone treatment, we identified two protein(s)-DNA complexes A and B for these GREs. The stable protein-DNA complex A (Fig. 6D, complex A, *lanes 2 and 10*) was produced as a result of GRE sequence-specific binding to GR, since the presence of anti-hGR antibody produced a supershift of the complex (Fig. 6E) and the addition of 50-fold excess of cold classic consensus GRE wild type (wt) or G6Pase GRE wt completely abolished it, while both classic consensus GRE mutant (mut) and G6Pase GRE mut, failed to do so (Fig. 6D, complex A in *lanes 3, 5, 11 and 12*, and complex A in *lanes 4 and 13*, respectively). In contrast, the complex B formed with radiolabeled classic consensus GRE wt did not indicate specific binding since both cold wild type and mutated GREs of the classic as well as the G6Pase type abolished it (Fig. 6D, complex B in *lanes 3, 4 and 5*). However, complex B formed with G6Pase GRE wt might represent specific binding of GR with this GRE, as G6Pase-GRE mut failed to abolish it (Fig. 6D, complex B in *lane 13*). Addition of recombinant LXRα/RXRα proteins to the nuclear extracts clearly decreased the band intensity of complex A produced with radiolabeled classic and wt G6Pase GREs (Fig. 6D, *lanes 6 to 7* and *lanes 14 to 15*). The decrease of GR association to its classic GREs was dose-dependent on the amounts of LXRα/RXRα (Fig. 6F, *upper panel*, compare *lanes 3 to 5 and 7*, and *lower panel*, compare *columns 3 to 5 and* 7). When GW3965 were added to the nuclear extracts, a significant decrease in GR binding to its classic radiolabeled GRE was observed both in the presence or absence of recombinant LXRα/RXRα (Fig. 6F, *upper panel*, compare *lanes 3 to 4; 5 to 6 and 7 to 8* and *lower panel*, compare *columns 3 to 4; 5 to 6; 7 to 8*). Furthermore, the incubation of classic GREs with recombinant LXRα/RXRα proteins in the absence of nuclear extracts developed a new protein(s)-DNA complex C, with similar migration properties as complex C in Fig. 6D (*lanes 9, 17 and 18*), indicating that LXRα and/or RXRα made a complex with radiolabeled classic and wt G6Pase GREs. Addition of GW3965 treatment enhanced the binding of LXRα/RXRα to the classic GRE (Fig. 6G, compare *lane 2 to 3*), whereas the presence of anti-hLXRα antibody resulted in a supershift of complex C (Fig. 6G, *lane 4*). These results indicate that LXRα/RXRα bind to GREs and decrease the association of GR to its GREs by competing with GR.

## Discussion

We demonstrated that ligand-activated LXRs regulated GR-induced transcriptional activity in a gene-specific fashion. This activity of the LXRs appeared to be more on the transactivating, and less on the transrepressing actions of glucocorticoids. This interaction was observed *in vivo* in the regulation of circulating glucose levels as an end-biological marker, as well as in the mRNA expression of G6Pase, a key enzyme in glucose metabolism, in both rat and mouse livers. In microarray analysis, the mutual effects between the LXRs and the GR were observed primarily from the direction of the former towards the latter. Consistent with the above findings, we demonstrated that LXRα/RXRα competed with GR for binding to consensus, as well as G6Pase and GILZ GREs *in vitro* and *in vivo*. These results were further confirmed by gel mobility shift assays in which LXRα/RXRα recombinant proteins were used to examine their interaction with classic or G6Pase GREs. This unexpected regulatory mechanism was previously observed with other nuclear receptors: RXRβ and its heterodimer partner peroxisome proliferator-activated receptor α interact with the estrogen response elements and regulate the expression of estrogen-responsive genes by competing with the estrogen receptor α for these DNA sequences [39], [40]. Although we examined only few GREs, we expect that LXR/RXR may bind GREs located in various glucocorticoid-responsive promoter regions to differentially regulate GR-induced transcriptional activity in a gene-specific fashion; this would explain at least in part the changes observed in our transcriptome analysis using microarrays.

LXRs are also known to repress actively some of their responsive genes, such as the inducible nitric oxide synthase (iNOS), by attracting corepressor NCoR [41], [42]. We examined the contribution of NCoR to LXR-mediated repression of GR-induced transcriptional activity using transient transfection-based reporter assays, but did not find an apparent cooperation between NCoR and the LXRs (data not shown). Thus, attraction of corepressors to GREs through LXRs/RXRs does not appear to be contributory to LXR-mediated repression of GR transcriptional activity.

GR-mediated transcriptional regulation is quite complex, with some effects exerted via direct binding of GR to GREs and others through protein-protein interactions with various transcription factors and/or cofactors [11]. Although the former correlates more with the transactivational than with the transrepressive effects of glucocorticoids, while the latter with the transrepressive rather than the transactivational activity of these steroids, this is not exclusive [43], [44]. We assume that such complex regulation of GR transcriptional activity is reflected in our microarray-based transcriptome analysis and our hypothesis is that activation of LXRs prevents primarily GRE-mediated transactivation and secondarily transrepression through competition between these receptors and the GRs for binding to GREs or interacting with other transcription factors. Indeed, the genes down-regulated by dexamethasone and further regulated by GW3965 may contain negative GREs through which the latter compound might have attenuated the suppressive effect of dexamethasone. Further study examining presence of negative GREs in the promoter regions of LXR/RXR-influenced glucocorticoid-responsive genes is necessary to verify this hypothesis.

During preparation of this manuscript, Patel *et al.* reported that LXRβ was required for some metabolic actions of glucocorticoids in the mouse liver, playing a supportive role in glucocorticoid-induced hyperglycemia and liver steatosis in LXRα/β^−/−^ mice [45]. Mechanistically, they demonstrated that dexamethasone-induced binding of GR to GREs was attenuated in a gene-specific fashion in the liver of LXRα/β^−/−^ mice [45], suggesting that endogenous LXRβ facilitates association of ligand-activated GR to GREs of some glucocorticoid-responsive promoters. In fact, before this manuscript was published, we proactively found that deletion of endogenous LXRα/β either by siRNA-mediated knockdown or by gene knockout attenuated dexamethasone-induced mRNA expression of the *PEPCK* gene. We, however, did not observe the positive effect of LXRα/β on GR-induced stimulation on G6Pase mRNA expression in contrast to the results demonstrated by this group, suggesting that this effect of endogenous LXRα/β on GR observed in the absence of LXR agonists is gene-specific. We do not know the exact mechanisms of this activity of endogenous, unliganded LXRα/β, but the complex promoter structure around the GREs of the PEPCK gene may be in part responsible [46], [47], [48]. However, once LXRs are activated by pharmacologic amounts of their ligands, LXRs suppressed GR-induced transcriptional activity of both the *G6Pase* and the *PEPCK* genes, possibly by inhibiting binding of this receptor to GREs through association with promoter regions of these genes. Taken together, our results provide important information on the regulation of GR actions by LXR ligands, while the results of Patel *et al.* and some of ours indicate the physiologic importance of LXRs on this receptor in the absence of ligands. Further intensive research will hopefully elucidate the molecular mechanism(s) underlying this positive to negative “switch” of the LXR activity on the GR in response to LXR ligands.

Glucocorticoids are commonly used for the treatment of a great variety of allergic, autoimmune and inflammatory diseases, such as asthma, rheumatoid arthritis, systemic lupus erythematosus and acute septic shock [12]. Numerous side effects are, however, associated with long-term and systemic use of pharmacologic doses of glucocorticoids, including increased gluconeogenesis, liposynthesis and insulin resistance, leading to development of metabolic syndrome, i.e., central obesity, carbohydrate intolerance, diabetes mellitus type 2 and dislipidemia, with consequent atherosclerosis and atherosclerosis-associated cardiovascular diseases [12]. Although, admittedly, this may appear simplistic, the glucocorticoid-related metabolic side effects are generally correlated with the transactivational properties of the GR, while its beneficial immunosuppressive effects are associated with its transrepressive actions [1], [7]. In our hands, LXRs strongly prevented glucocorticoid effects on glucose metabolism, e.g. on *G6Pase* mRNA expression, by repressing the transactivating activity of the GR, while no such effects were observed in the transrepressive actions of this steroid receptor on a NF-κB-responsive reporter gene in HCT116 cells (Fig. 2C). This specificity of the LXR effect on GR-induced transcriptional activity was recently confirmed by another group in the mouse spleen [45]. Thus, pharmacologic amounts of LXR agonists, such as GW3965, might be of benefit to patients receiving glucocorticoid treatment for allergic, autoimmune and inflammatory diseases, by attenuating the metabolic side effects of these steroids (Table 1). These results might also explain some conditions associated with simultaneous activation of LXR- and GR-mediated pathways. For example, patients with Cushing syndrome demonstrate both elevated levels of circulating glucocorticoids and hyperlipidemia [49], while subjects in acute or chronic stress or suffering from major depression, who demonstrate elevations of serum cortisol levels due to activation of the hypothalamic-pituitary-adrenal axis, develop components of the metabolic syndrome, such as visceral adiposity, hypertriglyceridemia, hypercholesterolemia and low HDL cholesterol [8]. Elevated circulating cortisol in these patients/subjects stimulates GR in target tissues, while elevated concentrations of circulating cholesterol and triglycerides, as well as their metabolites in local tissues, activate LXRs, possibly mitigating the effects of glucocorticoids. We hypothesize that activated GR increases glucose production by stimulating the transcriptional rate of G6Pase, and other enzymes, while the elevated LXR ligands suppress this GR effect by competing with GR for binding to GREs, forming a local counter regulatory protective loop. Activation of LXRs with pharmacologic use of their ligands appears to stimulate this intrinsic protective mechanism [16], [17]. Likewise, patients with anorexia nervosa who demonstrate elevated cortisol levels and reduced circulating cholesterol and triglycerides due to chronic stress and reduced food intake [50], may be associated with elevated GR transcriptional activity particularly in glucose metabolism due to attenuation of LXR-mediated repression on GR, which ultimately stimulates glucose production to counteract the hypoglycemia caused by reduced nutritional intake.

In our microarray analysis, dexamethasone and GW3965 regulated mRNA expression of a similar cluster of genes implicated in the inflammatory and immune response (Fig. 5A). These results are consistent with the previous report, which demonstrates GR and LXRs have an additive inhibition to a large number of functionally related inflammatory genes by intervening multiple, but distinct components of the Toll-like receptor 4 signaling pathway [51]. Thus, our results together with this previous report suggest that the simultaneous administration of LXR ligands may benefit the immunosuppressive effect of glucocorticoids in the treatment of allergic, autoimmune and inflammatory diseases. There is a caveat, however; some LXR agonists may also induce lipogenesis and increase triglyceride levels through induction of hepatic SREBP-1c gene expression [15]. Therefore, a challenge for the future will be to develop a new class of LXR ligands that will conserve a gene-specific effect on suppressing GR transcriptional activity, but in a selective manner, targeting LXRβ but not LXRα, as the latter plays an essential role in LXR agonist-mediated development of hypercholesterolemia [52].

In conclusion, we found that pharmacologic activation of LXRs suppresses GR-induced transcriptional activity by competing with GR for binding to GRE DNA sequences in a gene-specific fashion. Our results suggest that agonists for LXRs may be useful in preventing the adverse metabolic actions of glucocorticoids associated with chronic excess secretion, as in endogenous Cushing syndrome or chronic stress, or prolonged therapeutic use of pharmacologic doses [11], [12].

## Materials and Methods

### Plasmids and reagents

pCMX-hLXRα, -hRXRα, -mLXRβ and -mRXRα and pRShGRα, which express human (h) and mouse (m) LXRα and RXRα, and hGRα respectively, were all gifts from Dr. R.M. Evans (Salk Institute, La Jolla, CA). pCDNA3-6myc plasmids expressing the full-length human LXRα (amino acids 1–447) or its truncated forms consisting of amino acids 1–163, 1–127, 98–163 or 164–447 were generous gifts from Dr. D. Kardassis (Foundation of Research and Technology of Hellas, Crete, Greece). pCMX, which is a carrier plasmid for hLXRα, hRXRα, mLXRβ and mRXRα and was used as a negative control for these plasmids, and pGL4.73[*hRluc*/SV40], which expresses the renilla luciferase under the control of the simian virus 40 promoter, were purchased from Promega Corp. (Madison, WI). pMMTV-luc, pGILZ-luc and pPEPCK-luc plasmids, which express the firefly luciferase under the control of the indicated glucocorticoid-responsive promoters, were gifts from Drs. G.L. Hager (National Cancer Institute, Bethesda, MD), M. Pallardy (Institut National de la Santé et de la Recherche Medicale (INSERM), Paris, France) and D.K. Granner (Vanderbilt University Medical School, Nashville, TN), respectively. pRSV-RelA (p65) and pRSV-NF-κBI (p50), which respectively express p65 and p50 component of the nuclear factor-κB (NF-κB), were obtained from NIH AIDS Research and Reference Reagent Program (Germantown, MD). (κB)_3_-Luc, which expresses the firefly luciferase under the control of three κB-responsive elements (REs), was reported previously [53]. Recombinant hLXRα and hRXRα proteins were purchased from Active Motif (Carlsbad, CA). Anti-hGRα, anti-hLXRα, anti-hLXRα/β, anti-hRXRα, anti-human heat shock protein 4 (hHSP4) and rabbit control IgG antibodies were purchased from Santa Cruz Biotechnology Inc. (Santa Cruz, CA). Primers for hLXRα and β used in SYBR Green Real-Time PCR assays, siRNAs targeting hLXRα, hLXRβ or the control siRNA for luciferase GL2 were purchased from Qiagen (Valencia, CA). Dexamethasone, T0901317, GW3965 and 22-R-HC were purchased from Sigma-Aldrich (St Louis, MO).

### Cell cultures and transfections

Human hepatoma HepG2, human colon cancer HCT116 and the human cervical cancer HeLa cells were all purchased from American Type Culture Collection (Manassas, VA).

The human colon cancer HCT116 cells were cultured in McCoy's 5A medium supplemented with 10% fetal bovine serum (FBS) and 100 U/mL of penicillin and 100 µg/mL of streptomycin. The human cervical cancer HeLa and the human hepatoma HepG2 cells were cultured in Dulbecco's Modified Eagle's Medium (DMEM) with the same supplements. HCT116 cells do not express endogenous GR, while HeLa and HepG2 cells have the endogenous functional GR [20]. Cells were maintained in 5% CO_2_ at 37°C. For reporter assays, HCT116 cells were transfected with lipofectamine 2000™ (Invitrogen, Carlsbad, CA) with different amounts of the indicated plasmids, together with 0.5 µg/mL of pMMTV-Luc, pPEPCK-Luc, pGILZ-Luc or κB_3_-Luc vector and 0.1 µg/mL of pGL4.73[*hRluc*/SV40] in 12-well plates. Empty vectors were used to maintain the same amounts of transfected DNA. One day after the transfection, 10^−6^ M of dexamethasone and/or GW3965, T0901317 and/or 22-R-HC were added to the medium and the cells were further cultured for 24 hours. The cells were then harvested, and the firefly and renilla luciferase activities were measured using the dual-luciferase reporter assay system (Promega Corp.) in the GlowMax luminometer (Promega Corp.) according to the manufacturer's instructions. The relative luciferase activity (RLA) was calculated (light units from the firefly luciferase assay divided by the light units from the renilla luciferase assay) to correct for transfection efficiency.

HepG2 cells were transfected with the siRNAs indicated using the Nucleofector system (Reagent V and program T-28) (Amaxa GmbH, Cologne, Germany). Twenty-four hours after transfection, 10^−6^ M of dexamethasone (Dex) and/or GW3965 were added to the culture media. The following day, the cells were lysed and used for purification of total RNA using the RNeasy Mini Kit (Qiagen, Valencia, CA).

### SYBR Green-based real-time PCR

Total RNA was reverse transcribed into cDNA, and real-time PCR was performed in triplicate using the SYBR Green PCR Master Mix (Applied Biosystems, Foster City, CA) in a 7500 Real-time PCR system (Applied Biosystems), as previously described [54], [55]. Primer pairs used for the reactions are shown in Table 2. Obtained cycle threshold (C*t*) values of the target genes were normalized for those of RPLP0 or β-actin and their relative mRNA expression was demonstrated as fold induction over the baseline. The dissociation curves of primer pairs used showed a single peak and samples after PCR reactions had a single expected DNA band in agarose gel analysis (data not shown).

**Table 2:** Oligonucleotides Used in SYBR Green real-Time PCR Analysis and Gel Mobility Shift Assays.

Protein name		Primer sequence (5′ to 3′ orientation)
hG6Pase	Forward	TCATCTTGGTGTCCGTGATCG
	Reverse	TTTATCAGGGGCACGGAAGTG
hPEPCK	Forward	GAAAAAACCTGGGGCACAT
	Reverse	TTGCTTCAAGGCAAGGATCTCT
hGILZ	Forward	GATGTGGTTTCCGTTAAGC
	Reverse	CTCTCTCACAGCATACATCAG
hGRα	Forward	TGAAAATGGGTTGGTGCTTCTA
	Reverse	GACAAGAATACTGGAGATTTG
hABCG1	Forward	AGCATCATGAGGGACTCGGT
	Reverse	GGAGGCCGATCCCAATGT
hRPLP0	Forward	GAGGACCTCACTGAGATTCG
	Reverse	CTGGAAGAAGGAGGTCTTCTC
mG6Ppase	Forward	TTACCAAGACTCCCAGGACTG
	Reverse	GAGCTGTTGCTGTAGTAGTCG
mPEPCK	Forward	ATCTTTGGTGGCCGTAGACCT
	Reverse	GCCAGTGGGCCAGGTATTT
mβ-actin	Forward	AAGCTGTGCTATGTTGCTCTAGACT
	Reverse	CACTTCATGATGGAATTGAATGTAG
ratG6Pase	Forward	GGCTCACTTTCCCCATCAGG
	Reverse	ATCCAAGTGCGAAACCAAACAG
ratPEPCK	Forward	CCCAGACTAGAGATCCTGACAGAAT
	Reverse	GCACAACGCTCTTTTCTTTTACC
ratRPLP0	Forward	GAGAAGACCTCTTTCTTC
	Reverse	CAACATGTTCAGCAGTGTG
ratCCL9	Forward	AGTCTGAAGGCACAGCAAGGGC
	Reverse	CGGCCTGGTACACCCACCAC
ratCXCL12	Forward	TAGGCCACGCACGCAGCATC
	Reverse	GGCGTCTGACTCACACCTCTCAC
ratH6PD	Forward	GCGGTGGCTCAGATCCTGCC
	Reverse	CTGGCCCGACCTTCCGCATC
Classic GRE wt	5′	AGCTGGTACAAACTGTTCTAGCT
	3′	TCGACCATGTTTGACAAGATCGA
Classic GRE mut	5′	AGCTacgcgAgatgacgaTAGCT
	3′	TCGAtgcgcTctgctgctATCGA
G6Pase GRE wt	5′	GCACTGTCAAGCAGTGTGCCCAAGTTAATAATT
	3′	CGTGACAGTTCGTCACACGGGTTCAATTATTAA
G6Pase GRE mut	5′	GCACTacgcgGaAGgactaCCAAGTTAATAATT
	3′	CGTGAtgcgcCtTCctgatGGTTCAATTATTAA

G6Pase: glucose 6 phosphatase, PEPCK: phosphoenolpyruvate carboxykinase, GILZ: glucocorticoid-inducible leucine zipper protein, GR: glucocorticoid receptor, ABCG1: ATP-binding cassette sub-family G member 1, RPLP0: acidic ribosomal phosphoprotein P0, GRE: glucocorticoid response element, h: human, m: mouse, wt: wild type, mut: mutant – letters in lower cases indicate the mutated basis.

### Western blots

Proteins lysates and nuclear proteins from HepG2, HeLa cells or rat livers were prepared using the Nuclear Extract Kit (Active Motif), and were run on 4–20% SDS-PAGE gels. Separated proteins were blotted to nitrocellulose membranes. hGR, hLXRα, hLXRα/β and hHSP4 were visualized with anti-hGRα, anti-hLXRα, andti- hLXRα/β and anti-hHSP4 antibodies, respectively.

### Treatment of animals with dexamethasone and/or GW3965

*Ethics statement*: The following animal studies were approved by the NICHD Animal Care and Use Committee (protocol numbers: ASP 07-018 for the rat studies and ASP 09-009 for the mouse studies).

Wild type and LXRα/β knockout mice were generous gifts from Dr. D. Mangelsdorf (University of Texas Southwestern Medical Center, Dallas, TX). For each experiment, 4 male Sprague-Dawley rats (180–200 g), 4 wild type or 4 LXRα/β knockout mice were orally gavaged for three days with GW3965 prepared in 1% Tween 80, 0.5% hydroxypropyl methylcellulose, 20 mM Na_2_HPO_4_ and 20 mM NaH_2_PO_4_ (50 mg/kg animal). Another set of animals were also orally gavaged with the same volume of the vehicle control (1% Tween 80, 0.5% hydroxypropyl methylcellulose, 20 mM Na_2_HPO_4_ and 20 mM NaH_2_PO_4_). On the third day, GW3965 or its vehicle control was administered first to the animals followed by an intra-muscularly injection of dexamethasone (1.5 mg/kg) or physiologic saline in two animals from each group. Twenty-four hours after the injection, levels of blood glucose were measured in these rats using the Freestyle GlucoMeter (Abbott Laboratories, Abbott Park, IL) by sampling blood from their tails. The animals were then euthanized using CO_2_, and their livers were harvested for extraction and purification of total RNA and proteins.

### Microarray analysis

Five µg of total RNA purified from rat livers was used for producing probes with the One-Cycle Target Labeling and Control Reagents Kit (Affymetrix, Inc.). Rat Genome 230 2.0 Tiled Arrays (Affymetrix Inc.) were then labeled with the prepared probes, washed and stained in the Affymetrix working station (Affymetrix, Inc., Santa Clara, CA). Detailed data analysis steps were performed as previously described [20]. Briefly, all probe level annotations on the chip were verified before performing analysis by re-mapping to current Unigene sequences database (March 2006) and only those correctly mapped probes were used for analysis. Samples were examined using the PM – only method with MAS background signal subtraction [ref: www.bioconductor.org]. One sample Student *t*-test was performed based on comparisons of gene expression values (log ratio) of the same probe set among all replicates with a critical value of p≤0.05. Candidate genes were identified by using the z distribution by calculating the 95% cut off interval. The microarray data discussed in this publication are MIAME compliant and the raw data has been deposited in the Gene Expression Omnibus (GEO) database of the National Center for Biotechnology Information (www.ncbi.nlm.nih.gov/geo/) and are accessible through GEO Series accession number GSE29912.

### *In vitro* binding assays for evaluation of the GR/GRE association

HeLa cells were transfected with pCMX-hLXRα and -hRXRα or pCMX using the lipofectamine 2000™. Twenty-four hours after the transfection, cells were treated with 10^−6^ M of dexamethasone for 2 hours, and nuclear extracts were prepared using the Nuclear Extract Kit (Active Motif) in order to examine binding of GR to classic GREs just after translocation of the GR into the nucleus. *In vitro* binding assays for evaluating the association of GR to GREs were conducted by using the TRansAM™ GR Kit (Active Motif). Briefly, nuclear extracts were added to a 96-well ELISA plate, which was provided by the kit and had immobilized GRE oligonucleotides at the bottom of each well. Free oligonucleotide, which encoded wild type or mutant GREs, was added to some reactions to monitor specificity of the assay. The plate was then incubated first with anti-GRα antibody and second with horseradish peroxidase-conjugated antibody, and binding activity of GR to GREs was estimated by measuring absorbance at 450 nm with a reference wavelength of 655 nm in the Victor 3 (Applied Biosystems).

### Chromatin immunoprecipitation (ChIP) assays

ChIP assays were performed in HeLa, HepG2 and HCT116 cells. Briefly, cells were transfected with 0, 0.06 or 0.5 µg of pCMX-hLXRα and -hRXRα or pCDNA3-6myc pladmids using the lipofectamine 2000™. The cells were exposed to either 10^−6^ M of dexamethasone or vehicle for 6 hours, and were subsequently fixed, DNA and bound proteins were cross-linked for 10 min with 4% formaldehyde, and ChIP assays were performed by co-immunoprecipitation of the DNA/protein complexes with anti-hGRα, -hLXRα or -hRXRα antibodies or rabbit control IgG (Santa Cruz Biotechnology, Inc.). We employed 6-hour incubation with dexamethasone, as we found that this time point is sufficient to detect accumulation of proteins on GREs in our assay system (data not shown). The promoter region (−257 to −39) of the endogenous *G6Pase* gene, which contains three functional GREs [56], and that (−1341 to −1209) of the endogenous *GILZ* gene promoter that encloses one tandem GRE [24], were amplified from the prepared DNA samples using the specific primer pairs (G6Pase: Forward: 5′-CAGACCCTTGCACTGCCAAGAAGCATG-3′ and Reverse: 5′-TATCCAGTATTCAGGTCAACCCAGCCC-3′, and GILZ: Forward: 5′-CCTTAACTTCATCCAAACTG-3′ and Reverse: 5′-CACCAGAAGGAGCAAGAG-3′) in the SYBR Green real-time PCR using the SYBR Green PCR Master Mix and a 7500 Real-time PCR System. Obtained C*t* values of ChIP samples were normalized for those of corresponding inputs, and their relative precipitation was expressed as fold precipitation above the baseline.

### Gel mobility shift assays

Double stranded oligonucleotides for classic or G6Pase GREs [56] (Table 2) were radiolabeled with [γ-^32^P]dCTP using the T4 polynucleotide kinase (Invitrogen). Nuclear extracts from HeLa cells (10 µg) or hLXRα/hRXRα recombinant proteins were preincubated for 10 min at 4°C in the binding buffer [10 mM HEPES pH 8.0, 0.1 mM EDTA, 2 mM DTT, 50 mM NaCl, 5 mM MgCl_2_, 50 mM KCl, 4 mM spermidine, 4% Ficoll, and 1 µg poly(dI-dC)] in the presence or absence of an excess amount of unlabeled oligonucleotides encoding GRE wt, GRE mut, G6Pase wt or G6Pase mut (Table 2). The corresponding radiolabeled oligonucleotide probes (1×10^5^ cpm) were then added to each sample, and the binding reaction was allowed to proceed at room temperature for 15 min in the presence or absence of anti-hGR or anti-hLXRα antibody. Nuclear proteins bound to radiolabeled oligonucleotide probes were separated from free probes on 6% DNA retardation gel (Invitrogen) at 100 V for 50 min, and the gel was dried and exposed to Biomax MR Film (Eastman Kodak, St. Louis, MO) at −80°C.

### Statistical analysis

All experiments were performed with duplicate or triplicate samples and were repeated at least twice. Statistical analysis was carried out by ANOVA, followed by Student's *t* test with Bonferroni correction for multiple comparisons or unpaired Student *t* test with a two-tailed *P* value.

## Supporting Information

Table S1 **GW3965 altered mRNA expression of 77 dexamethasone resistant genes.**
(DOC)

Table S2 **GW3965 altered mRNA expression of 45 (∼6%) genes down-regulated by dexamethasone.**
(DOC)

Table S3 **GW3965 altered mRNA expression of 66 (∼9%) genes up-regulated by dexamethasone.**
(DOC)
