# Supplementary material for: Liver X Receptors Regulate the Transcriptional Activity of the Glucocorticoid Receptor: Implications for the Carbohydrate Metabolism
Source: PLoS One. 2012 Mar 22;7(3):e26751. doi: 10.1371/journal.pone.0026751 (PMC3310817; doi:10.1371/journal.pone.0026751)
Supplement: Table S1 — GW3965 altered mRNA expression of 77 dexamethasone resistant genes. (DOC) [file pone.0026751.s001.doc]

| **Gene symbol** | **Gene access ID** | **Log ratio (GW)** |
| --- | --- | --- |
| Cyp7a1 | NM_012942 | -3.61933 |
| Dnpep | AI412298 | -2.16479 |
| --- | BE116572 | -2.11189 |
| Mlc1 | AI228307 | -1.56332 |
| Itga1 | NM_030994 | -1.5455 |
| Tspyl4 | BI281738 | -1.52657 |
| Bcl6 | AI237606 | -1.49129 |
| Lyz2 | L12458 | -1.37293 |
| Cyp26b1 | BF397093 | -1.35793 |
| --- | BF522747 | -1.30927 |
| --- | BF386770 | -1.27214 |
| --- | BF395080 | -1.26041 |
| --- | BF564309 | -1.24741 |
| --- | AI012869 | -1.23948 |
| Pde4b | AF202733 | -1.21273 |
| Cyp39a1 | BE115944 | -1.17362 |
| Tmem166 | AW251313 | -1.16945 |
| RGD1562844 | BI274644 | -1.1392 |
| --- | AA818342 | -1.12993 |
| --- | BE103947 | -1.07948 |
| --- | BI288131 | -1.07169 |
| Paip1 | AA963084 | -1.07154 |
| Tm7sf2 | BM390364 | -1.06487 |
| Lpl | NM_012598 | -1.06102 |
| --- | AW526127 | -1.02915 |
| --- | BG377636 | -1.02506 |
| Pou2f1 | AW528459 | -1.02215 |
| Tmem55a | BI293584 | 1.000166 |
| --- | AI228346 | 1.022787 |
| --- | BF391308 | 1.023337 |
| Pxmp4 | BG664011 | 1.024861 |
| LOC100134871 /// LOC689064 /// MGC72973 | X05080 | 1.027741 |
| Gbp2 | NM_133624 | 1.029624 |
| --- | AA964492 | 1.046967 |
| --- | BF400811 | 1.067354 |
| Lox | BI304009 | 1.080402 |
| Cd14 | NM_021744 | 1.106098 |
| Egr2 | NM_053633 | 1.108837 |
| --- | AI172110 | 1.123773 |
| Grem2 | AA817956 | 1.12815 |
| --- | AI101500 | 1.129672 |
| Hspa1b | BI278231 | 1.132294 |
| Lss | BM390574 | 1.159304 |
| LOC100134871 /// LOC689064 /// MGC72973 | BI287300 | 1.165211 |
| RGD1565709 | BI279202 | 1.170188 |
