# Supplementary material for: Liver X Receptors Regulate the Transcriptional Activity of the Glucocorticoid Receptor: Implications for the Carbohydrate Metabolism
Source: PLoS One. 2012 Mar 22;7(3):e26751. doi: 10.1371/journal.pone.0026751 (PMC3310817; doi:10.1371/journal.pone.0026751)
Supplement: Table S2 — GW3965 altered mRNA expression of 45 (∼6%) genes down-regulated by dexamethasone. (DOC) [file pone.0026751.s002.doc]

| **Gene symbol** | **Gene access ID** | **Log ratio (Dex)** | **Log ratio (Dex+GW3965)** |
| --- | --- | --- | --- |
| *Hsd17b2* | NM_024391 | -4.194693029 | -2.947419823 |
| *Prm1* | AI144723 | -4.02495553 | -2.820465638 |
| *Slc34a2* | NM_053380 | -3.854386123 | -2.221751178 |
| *Inmt* | AI232716 | -3.485257813 | -2.467463668 |
| *Stac3* | BM385735 | -3.412354653 | -2.046425523 |
| *Unkown* | BE098538 | -3.347054075 | Not significant |
| *Unkown* | L22655 | -3.060067354 | -1.801219384 |
| *Lum* | NM_031050 | -2.676206943 | -1.261642808 |
| *Dusp6* | AI602811 | -2.605782664 | Not significant |
| *Unkown* | BG374683 | -2.592928052 | Not significant |
| *Sult1c2* | BI300997 | -2.590304098 | -1.484687354 |
| *Dusp6* | NM_053883 | -2.474271415 | Not significant |
| *Ccnd1* | BI295861 | -2.435907952 | -1.131071448 |
| *Unkown* | BM384088 | -2.313596382 | Not significant |
| *Gemin6* | BF283004 | -2.206063471 | Not significant |
| *Unkown* | BF282112 | -2.113511535 | Not significant |
| *Akr1d1* | AI028867 | -2.047432143 | Not significant |
| *Cish* | AF065161 | -2.0082525 | Not significant |
| *Myc* | NM_012603 | -1.990609251 | Not significant |
| *Igha_mapped* | AI412189 | -1.971001214 | Not significant |
| *Ier5* | BF285187 | -1.924877486 | Not significant |
| *Cyp2c12* | NM_031572 | -1.924204094 | Not significant |
| *Fabp7* | NM_030832 | -1.922017281 | Not significant |
| *Unkown* | AI411141 | -1.912372639 | Not significant |
| *Dusp6* | AI231350 | -1.826384023 | Not significant |
| *Unkown* | AA925921 | -1.708534957 | Not significant |
| *Slc10a2* | NM_017222 | -1.684272618 | Not significant |
| *Pbsn* | NM_019125 | -1.676598102 | Not significant |
| *Foxa2* | NM_012743 | -1.539579568 | Not significant |
| *Socs2* | NM_058208 | -1.526135623 | Not significant |
| *Slco1a4* | U95011 | -1.525455774 | -2.608706763 |
| *Rgs3* | NM_019340 | -1.432762628 | Not significant |
| *Cish* | BI291842 | -1.430872378 | Not significant |
| *Tc2n* | BI302544 | -1.313978676 | Not significant |
| *Unkown* | AA818166 | -1.312221159 | Not significant |
| *Unkown* | BF394158 | -1.308266182 | Not significant |
| *Atpif1* | AF368860 | -1.26912032 | Not significant |
| *Unkown* | BF409092 | -1.256946433 | -3.225594345 |
| *Hhex* | NM_024385 | -1.197796177 | Not significant |
| *Slco1a4* | NM_131906 | -1.141221395 | -2.341291334 |
| *Ubxd6* | BF394199 | -1.088923519 | Not significant |
| *Unkown* | BF283311 | -1.068224774 | Not significant |
| *Egfr* | M37394 | -1.052289224 | -2.385874398 |
| *Unkown* | AW526964 | -1.048641596 | Not significant |
| *Unkown* | BI303527 | -1.007956762 | Not significant |
