# Supplementary material for: Liver X Receptors Regulate the Transcriptional Activity of the Glucocorticoid Receptor: Implications for the Carbohydrate Metabolism
Source: PLoS One. 2012 Mar 22;7(3):e26751. doi: 10.1371/journal.pone.0026751 (PMC3310817; doi:10.1371/journal.pone.0026751)
Supplement: Table S3 — GW3965 altered mRNA expression of 66 (∼9%) genes up-regulated by dexamethasone. (DOC) [file pone.0026751.s003.doc]

| **Gene symbol** | **Gene access ID** | **Log ratio (Dex)** | **Log ratio (Dex+GW3965)** |
| --- | --- | --- | --- |
| *Rnf31* | BF411972 | 1.009217099 | 2.143310557 |
| *Unkown* | BF418338 | 1.033267476 | Not significant |
| *Tat* | M18340 | 1.040419475 | Not significant |
| *Unkown* | BI297236 | 1.06156153 | Not significant |
| *Fzd7* | AI010048 | 1.06581449 | Not significant |
| *Enc1* | AI179988 | 1.067543553 | Not significant |
| *Dhx58* | AA818849 | 1.09059154 | 1.672559997 |
| *LOC679623* | AA923865 | 1.165587573 | 2.748619191 |
| *Parp12* | BI285978 | 1.19844285 | 2.259846474 |
| *Unkown* | AA957343 | 1.249189716 | Not significant |
| *Ppcs* | AI231606 | 1.273314293 | Not significant |
| *Rsad2* | AI409634 | 1.301231362 | 2.699695036 |
| *Fmo2* | BM389350 | 1.302707396 | Not significant |
| *Coq10b* | AI228596 | 1.310439219 | Not significant |
| *Trp53inp1* | AA946371 | 1.352680471 | Not significant |
| *Cxcl10* | U22520 | 1.35365368 | 2.991056325 |
| *G6pc* | NM_013098 | 1.35523708 | Not significant |
| *Unkown* | BF399517 | 1.361561239 | Not significant |
| *Zfp189* | AI407872 | 1.38191348 | Not significant |
| *Slain2* | AI407974 | 1.397630472 | Not significant |
| *Stat1* | NM_032612 | 1.406285367 | 2.490605829 |
| *Unkown* | BF401102 | 1.41971819 | Not significant |
| *G6pc* | U07993 | 1.473269685 | Not significant |
| *RGD1563091* | AA998964 | 1.527012176 | 2.753052237 |
| *Atp1b1* | AI232036 | 1.527243023 | Not significant |
| *Ppp1r3c* | BM390827 | 1.625573045 | Not significant |
| *Unkown* | BF419844 | 1.659889399 | Not significant |
| *Herc6* | AA943147 | 1.679164111 | 3.479199101 |
| *Ppp1r3c* | AW530361 | 1.696687031 | Not significant |
| *Ctgf* | NM_022266 | 1.758598303 | Not significant |
| *LOC684871* | AI230625 | 1.764044085 | Not significant |
| *H28* | AA819629 | 1.791008484 | 3.192288665 |
| *Unkown* | BF402416 | 1.798214872 | Not significant |
| *Mx2* | NM_134350 | 1.800862865 | 2.894564612 |
| *Herpud1* | NM_053523 | 1.833948255 | Not significant |
| *Unkown* | BE107649 | 1.846475129 | Not significant |
| *Unkown* | AA944136 | 1.910475486 | Not significant |
| *Unkown* | BE103875 | 1.924568366 | 1.638502354 |
| *Mtmr7* | BF392344 | 2.026375992 | Not significant |
| *Unkown* | AI407719 | 2.030225415 | Not significant |
| *Ddx60* | BI303853 | 2.031249236 | 3.440376177 |
| *Unkown* | AI169140 | 2.150612241 | Not significant |
| *Got1* | D00252 | 2.21325237 | Not significant |
| *Lmod2* | AI453854 | 2.243206877 | Not significant |
| *Unkown* | BI288533 | 2.255109833 | Not significant |
| *Cdkn1a* | AI010427 | 2.333514602 | 1.08259093 |
| *Cdkn1a* | U24174 | 2.354387169 | Not significant |
| *Unkown* | AA859079 | 2.373904499 | 1.070424096 |
| *Unkown* | BF403932 | 2.437762646 | Not significant |
| *Usp2* | AF106659 | 2.454488297 | 1.35782888 |
| *Irf7* | BF411036 | 2.589377931 | 3.672509376 |
| *Unkown* | BE113272 | 2.850543762 | Not significant |
| *Mx1* | X52711 | 2.875019838 | 4.270111075 |
| *Fkbp5* | BI284255 | 2.890421479 | 1.591723672 |
| *Lpin1* | BM385286 | 3.05264174 | 1.497669549 |
| *Fkbp5* | AW534837 | 3.181121633 | 1.779844852 |
| *Igfbp1* | NM_013144 | 3.193986109 | 1.679969087 |
| *Unkown* | BG378238 | 3.294619001 | 1.67856327 |
| *Mx2* | NM_017028 | 3.411746686 | 5.130415585 |
| *RGD1560784* | AI136882 | 3.429977859 | 1.8109648 |
| *Sds* | NM_053962 | 3.521152059 | 1.778875641 |
| *Rbp7* | BI283223 | 3.57526708 | 2.288822928 |
| *G1p2* | BE096523 | 3.769362464 | 5.279423599 |
| *Cyp1a1* | X00469 | 4.004491576 | Not significant |
| *Zfp354a* | NM_052798 | 4.511834989 | 1.820515408 |
| *Fam111a* | AA799328 | 5.814874638 | Not significant |
